# A primary care alternative to a hospital-based approach to COVID-19 in India

**DOI:** 10.7189/jogh.10.020346

**Published:** 2020-12

**Authors:** Amita Sudhir, Nachiket Mor

**Affiliations:** 1 University of Virginia Department of Emergency Medicine, Charlottesville, Virginia, USA; 2 The Banyan Academy of Leadership in Mental Health, Bangalore, Karnataka, India

India, like many low- and middle-income countries (LMICs), lacks significant hospital and emergency transportation infrastructure. As the COVID-19 pandemic spreads in India, large urban centres are already starting to become overwhelmed as they did in higher income countries. With only 0.7 hospital beds per 1000 population [1], the system stands to be overloaded, in great part by patients who require no intervention or only require oxygen support and little other intervention, because the focus in treating COVID-19 has been nearly entirely hospital-based in India, just as elsewhere in the world. India, however, has the primary care infrastructure to offer a different solution, one that is based in the community, utilising the skills held by primary care providers, and the resources available to them.

In India, as in the rest of the developing world, the number of hospital beds and ventilators per 1000 population is quite low as compared to High Income Countries (HICs), where, despite the greater availability of beds, resources were quickly overwhelmed. A significant positive correlation between mortality and the rising number of cases causing health systems to be overwhelmed has been observed, and this is particularly concerning for LMICs [2]. Given this reality, every effort should be made to minimize the burden on hospitals, to allow them to focus on caring for patients who require critical care interventions such as non-invasive positive pressure ventilation, mechanical ventilation, and blood pressure support.

Most cases of COVID-19 are mild and do not require hospital based care. In the first large study of the breakdown of COVID-19 patients conducted by the Chinese Centers for Disease Control in Wuhan, China, 81 percent had disease that was classified as mild. 14 percent had disease that was classified as severe, which was defined as hypoxia, dyspnea, or worsening lung infiltrates. There were no deaths in either the mild or severe groups. And only 5 percent were defined as critical, with respiratory failure, septic shock, or multiorgan dysfunction, with all deaths in this group [3]. The vast majority of COVID patients, therefore, can be managed in an outpatient setting, and safely sent home with isolation instructions and return precautions after a diagnosis is made, or even pending a diagnosis. Of the patients classified as severe, many can be managed with simple interventions such as nasal cannula oxygen to reverse hypoxia, and fluid resuscitation if needed. These interventions do not require a trained hospitalist or intensivist to administer, nor do they necessarily need to be administered in a traditional hospital setting. Primary care providers with a knowledge of basic medicine can correct hypoxia and make a determination of when to escalate to higher levels of care when interventions are not working.

Of the available interventions to correct hypoxia, which is often the primary reason for hospital admission in COVID-19 patients, the simplest is oxygen by nasal cannula. This can be administered at any facility with the ability to keep patients overnight or for multiple days, but other than oxygen concentrators or cylinders, nasal cannulas, and simple pulse oximeters, no specialized equipment or advanced monitoring is needed. Concentrators have the advantage of not relying on a continuous supply chain and do not need to be frequently replaced; oxygen cylinders have the advantage of not needing a reliable power supply to operate [4]. The source of oxygen can be tailored to the constraints of the setting.

Many patients will respond to these interventions and can be kept at the initial treatment site until symptoms improve. If possible, arrangements could also be made for patients with appropriate home support and lower oxygen requirements to be discharged with oxygen delivery devices and strict return precautions. Patients who are deemed too sick to go home or who lack the necessary care support could be kept at the facility. IV access could be established in case of deterioration and the need for fluid resuscitation. The assessment of these patients and a decision about whether they need to be transferred to a higher level of care can be made by primary care physicians even without subspecialty training, using easy to measure parameters: sPO_2_, blood pressure, heart rate, and respiratory rate. The development of protocols and guidelines would aid these physicians in providing standardised care. Other low cost and simple interventions such as steroids [5], antibiotics for secondary pneumonia, bronchodilators, and prophylaxis against venous thromboembolism can also be administered in these facilities. Awake proning is another simple but beneficial intervention that can be applied with minimal provider training, and does not require a higher level of care to execute [6].

Remote expert assistance via telemedicine could be made available to these providers for cases in which the need for hospitalization or transfer to a higher level of care is unclear. We have created a simple oxygen protocol that can be modified or added to, allowing it to be used in a variety of different settings [7]. If clinic spaces are unavailable to establish a facility, any large space that allows for appropriate distancing between patients, has toilet facilities, and adequate ventilation, can be repurposed as an oxygen centre. Staff would undergo a brief, basic training in implementing the protocol.

India and most LMICs have the resources to approach this problem using existing primary care providers and clinics. While hospital beds and emergency transportation infrastructure are scarce commodities in these countries, a strong community-based infrastructure exists, consisting of community organizations and local nonprofits. India, for example, has a large network of primary care and community health facilities: 25 000 primary health centers and 5300 community health centers owned by the government and a much larger number in the private and the non-profit sectors [8]. These organizations could be harnessed in the COVID-19 effort as follows: identifying the most vulnerable and guiding families on how to keep them protected; teaching home-care guidelines, even for sick patients, to avoid hospitalization [9]; mapping out a network of primary care providers, (public, private and non-profit); assigning each family a provider who would be responsible for testing and evaluation should anyone become sick, to avoid a trip to the hospital; providing resources to these Primary care providers for managing all but the sickest of patients, including training and education on the basic management of COVID patients. The availability of such physicians and nurses, including those with formal undergraduate or graduate qualifications in Allopathic, Ayurvedic, Unani, and Siddha medicine and surgery, and the availability of maternity homes and mini hospitals [8] are valuable potential assets in the fight against COVID.

**Figure:**
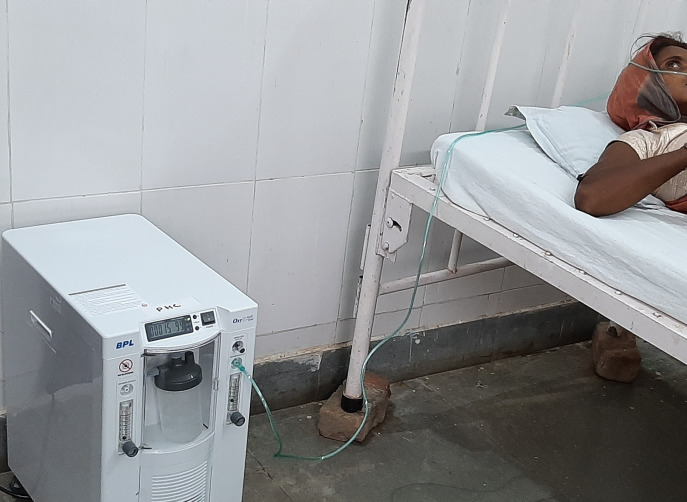
Photo: use of an oxygen concentrator in primary health care (Basic Healthcare Services https://bhs.org.in).

The issues impacting coronavirus care in India are common to many developing countries and this framework for tackling it as a primary care problem could be applied to many other health care systems across the world. Much of the published literature on COVID in LMICs focuses on prevention, and while this is of paramount importance, recent events show us that this is clearly not enough. The solution of using primary care facilities and providers is adaptable to either a public or private setting, depending on the resources available in any given country. The investment in infrastructure needed is not only relatively small (the cost of oxygen concentrators and provider training), but also represents an investment in items that will have continued utility post-COVID (if we may look forward to such a time) – widespread availability of oxygen, a life-saving and temporizing intervention, and training for providers in oxygen delivery, an important aspect of basic emergency care. Oxygen concentrators are portable, and once the need for using them for COVID patients has passed, they can be repurposed to ambulances, clinics, and even home health settings for a host of other diseases causing hypoxia. If oxygen cylinders are used, maximizing that supply chain will continue to be of benefit for the treatment of respiratory illnesses for years to come. If the pandemic drags on for months to years, oxygen centers could also become screening points for other diseases likely to be neglected during the pandemic, such as tuberculosis, or foci for immunization campaigns for ongoing endemic or epidemic illnesses beyond COVID.

Frenk and colleagues describe the need for a “diagonal” approach in building health systems. While the focus is on the “vertical” strategies needed to address the immediate crisis, their implementation also keeps in mind the needs of the overall “horizontal” health system [10]. The solution we outline meets exactly these two criteria and has the potential to help LMICs respond at a sufficient scale to the current crisis but also to leave behind a considerably strengthened health care system.
